# RD-GuideNet: A Depth-Guided Framework for Robust Detection, Segmentation, and Temporal Tracking of White Button Mushrooms

**DOI:** 10.3390/s26061935

**Published:** 2026-03-19

**Authors:** Namrata Dutt, Daeun Choi, Yiannis Ampatzidis, Won Suk Lee, Sanjeev J. Koppal, Xu Wang

**Affiliations:** 1 Department of Agricultural and Biological Engineering, Gulf Coast Research and Education Center, Institute of Food and Agricultural Sciences, University of Florida, Wimauma, FL 33598, USA; namrata.dutt@ufl.edu (N.D.); xuwang1@ufl.edu (X.W.); 2 Department of Agricultural and Biological Engineering, Southwest Florida Research and Education Center, Institute of Food and Agricultural Sciences, University of Florida, Immokalee, FL 34142, USA; i.ampatzidis@ufl.edu; 3 Department of Agricultural and Biological Engineering, Institute of Food and Agricultural Sciences, University of Florida, Gainesville, FL 32603, USA; wslee@ufl.edu; 4 Department of Electrical and Computer Engineering, University of Florida, Gainesville, FL 32603, USA; sjkoppal@ece.ufl.edu

**Keywords:** automated harvesting, depth fusion, depth-guided computer vision, instance segmentation, precision agriculture, RGB-D, temporal tracking, 3D shape analysis

## Abstract

**Highlights:**

- Developed RD-GuideNet, a novel depth-guided segmentation and tracking framework integrating RGB and depth information for automated mushroom perception.
- Achieved F1-score of 0.93 for segmentation and 92.7% tracking consistency, outperforming YOLOv8 (F1-score = 0.88) and YOLOv11 (F1-score = 0.86) in geometric accuracy.
- Demonstrated that depth-based geometric reasoning produces sharper, more physically consistent boundaries than appearance-based deep learning models.
- Identified that combining RD-GuideNet’s geometric precision with deep learning’s high recall can enhance perception reliability for autonomous mushroom harvesting.

**Abstract:**

Mushroom farms in the United States continue to face persistent labor shortages, especially during the harvesting of white button mushrooms (*Agaricus bisporus*) which requires selective picking by skilled workers. This study addresses this challenge by developing a depth-guided computer vision framework for automated mushroom detection, segmentation, and tracking to support timely harvest decisions, providing the foundation needed to support selective and timely robotic harvesting. The specific objectives of the study were to (1) develop a novel image-processing algorithm (RD-GuideNet) that integrates RGB and depth images for accurate detection and segmentation of mushrooms; (2) implement a custom depth-guided tracking algorithm to preserve mushroom identities across sequential frames; (3) compare the performance of RD-GuideNet against state-of-the-art deep learning models, YOLOv8 and YOLOv11, focusing on segmentation and tracking accuracies. The proposed RD-GuideNet achieved an F1-score of 0.93 for segmentation, outperforming YOLOv8 (0.88) and YOLOv11 (0.86), and produced sharper, more geometrically consistent boundaries that closely followed true mushroom cap contours. Its tracking consistency reached 92.7%, compared to YOLOv8 (95.3%) and YOLOv11 (94.6%). Although slightly lower, RD-GuideNet maintained high temporal consistency across dense mushroom beds. These results suggest that depth-based geometric reasoning and deep learning approaches exhibit complementary strengths in dense production scenes. Combining the two may further enhance detection reliability and shape fidelity, supporting high-precision perception for autonomous mushroom harvesting. A comprehensive quantitative evaluation of such a hybrid framework will be investigated in future work.

## 1. Introduction

### 1.1. Background and Motivation

White Button Mushroom (*Agaricus bisporus*) is one of the most widely cultivated mushrooms globally for its flavor and nutritional value [1]. In the United States, production occurs year-round in a controlled environment, with Pennsylvania and California contributing the majority of national output [2]. During the 2024–2025 season, U.S. growers produced approximately 0.3 million tons of white button mushrooms with an estimated economic value of nearly $1 billion [3]. Despite its economic significance, the mushroom industry has faced multiple challenges, including pests, diseases, and labor shortages. Among those, the persistent labor shortage continues to constrain production because harvesting relies heavily on skilled manual workers. White button mushrooms require selective manual harvesting, where mature mushrooms are handpicked while immature ones continue to grow. In current practice, workers evaluate maturity by observing multiple visual cues such as cap diameter, flattening, color, and stalk elongation [4,5,6,7]. In addition, workers should perform thinning to maintain optimal spacing between mushrooms during growth, which further increases the labor burden [8]. These operations are repetitive, physically demanding, and increasingly difficult to sustain as labor availability declines. As a result, there is growing interest in robotic harvesting systems that can assist manual operations in commercial mushroom production.

### 1.2. 2D Vision Approaches and Limitations

Recent advancements in agricultural robotics have shown the potential for automating these repetitive and skill-intensive tasks [9,10,11]. However, successful robotic harvesting depends critically on reliable computer vision systems capable of accurate mushroom detection and segmentation, particularly in densely populated beds where mushrooms grow in close proximity. Early studies primarily relied on traditional, feature-based image processing algorithms that operated on two-dimensional imagery, which limited robustness when caps were touching or partially occluded. Van De Vooren et al. [12] used morphological feature descriptors to differentiate mushroom cultivars, achieving 80% accuracy, but their reliance on 2D binary silhouettes led to boundary fusion and shape distortion when caps touched or were partially hidden. Zhou et al. [13] applied edge detection and geometric measurements to locate mushrooms, reporting an accuracy of 84%. Nobel et al. [14] investigated factors such as mushroom density, cap size, and orientation affecting harvesting performance and achieved a picking rate of 74%. Despite these contributions, all of these approaches remained constrained by 2D imagery, limiting segmentation reliability and harvesting performance in dense mushroom beds.

### 1.3. Depth/3D Sensing Approaches for Improved Geometry

To address the geometric ambiguity of 2D imagery, depth-based approaches have been explored to improve boundary localization and shape measurement. Ji, Sun, Zhao et al. [6] used depth images combined with Hough Transform to measure cap diameter, achieving 92.3% accuracy, demonstrating the value of 3D geometric cues for distinguishing individual caps. Retsinas et al. [15] extended this idea by using a 3D point cloud for pose estimation and segmentation, reporting a mean average precision of 99.8%. These studies confirmed that depth information can enhance geometric accuracy, particularly for overlapping or visually similar caps. However, many depth-based pipelines relied on multiple hand-tuned processing stages and were sensitive to noise, shallow height differences, and cap overlap, which often limited boundary precision under practical conditions.

### 1.4. Deep Learning Approaches

More recently, deep learning approaches have improved robustness by learning features directly from data and have been increasingly applied to mushroom detection and segmentation. For example, probabilistic neural networks combined with GrabCut were explored for mushroom segmentation, although performance was sensitive to dataset imbalance [16]. Lee et al. [17] integrated RGB images with 3D point clouds using deep learning frameworks such as Faster R-CNN to estimate mushroom size and maturity, achieving moderate recall (82%) at the cost of substantial computational complexity. YOLO-based models and instance-segmentation frameworks have also been applied to mushroom detection and classification, with performance often degrading for small, densely clustered, or partially occluded mushrooms, with additional architectural modifications required to improve attention to small objects [18,19]. While these learning-based approaches provide strong detection coverage and generalization across visual conditions, they typically rely on large, annotated datasets and produce smoother, appearance-driven masks that do not always align tightly with true cap boundaries. For robotic harvesting, however, precise geometric delineation of individual caps is critical to avoid collisions in dense beds and to support reliable downstream measurements. These considerations motivate depth-guided, geometry-driven alternatives that explicitly prioritize boundary accuracy.

### 1.5. Need for Temporal Tracking

Despite these advances, most existing studies focus on single-frame detection and segmentation and do not explicitly address the challenge of tracking mushrooms through time. This limitation is particularly important for robotic harvesting systems, because mushroom maturity is not defined by a static appearance but by the onset and progression of geometric changes, such as cap flattening. In dense beds, spatial constraints often suppress diameter expansion, allowing mushrooms to mature without substantial increases in size. Under these conditions, maturity cannot be reliably inferred from a single snapshot; instead, it must be determined by observing how individual caps evolve over time. Temporal tracking, therefore, becomes a necessary component. By linking detections across sequential frames, tracking enables stable identification and provides a foundation for growth-aware decision-making in automated harvesting systems.

### 1.6. Prior Work on Temporal Tracking

Only a limited number of studies have addressed mushroom tracking. For example, Simon et al. [20] proposed a semi-automatic pipeline for detecting and tracking mushrooms in time-lapse image sequences by modeling caps as circles and propagating detections backward from the final frame to earlier images, primarily to support efficient ground-truth generation. Similarly, Nuwayhid et al. [21] applied Mask R-CNN with IoU-based association to track oyster mushroom clusters and estimate growth through pixel-area changes. Ground-truth was obtained using manual annotations of mushroom (or cluster) boundaries, and the size measurements are computed directly from the resulting segmentation masks using mask area in pixels. However, both studies relied exclusively on 2D RGB time-lapse imagery. As a result, depth-informed tracking, particularly approaches that exploit 3D geometric cues to support robust tracking and boundary-aware analysis in dense mushroom beds, remains largely unexplored in the literature.

### 1.7. Study Objectives

To address these limitations, this study proposes a Depth-Guided Image-Processing Pipeline for Mushroom Detection and Segmentation (RD-GuideNet), a depth-guided detection, instance segmentation, and tracking framework designed for dense white button mushroom beds. RD-GuideNet explicitly leverages depth gradients and spatial constraints to prioritize geometric boundary accuracy, while maintaining temporal consistency across frames to support downstream harvesting decisions. By integrating depth-guided segmentation with multi-frame tracking, the proposed framework aims to bridge the gap between high-coverage detection and the precise, growth-aware perception required for practical robotic mushroom harvesting. The specific objectives were to: (1) develop a depth-based image-processing algorithm (RD-GuideNet) for accurate detection and segmentation of white button mushrooms using integrated RGB and depth data; (2) design a custom depth-guided multi-frame tracking algorithm to maintain consistent mushroom identities across sequential frames and reduce errors from occlusion or co-joined caps; (3) quantitatively evaluate the proposed RD-GuideNet and tracking algorithm against state-of-the-art deep learning models (YOLOv8 and YOLOv11) in terms of detection, segmentation, and tracking performance.

## 2. Materials and Methods

### 2.1. Data Collection and Experimental Setup

**Figure 1:**
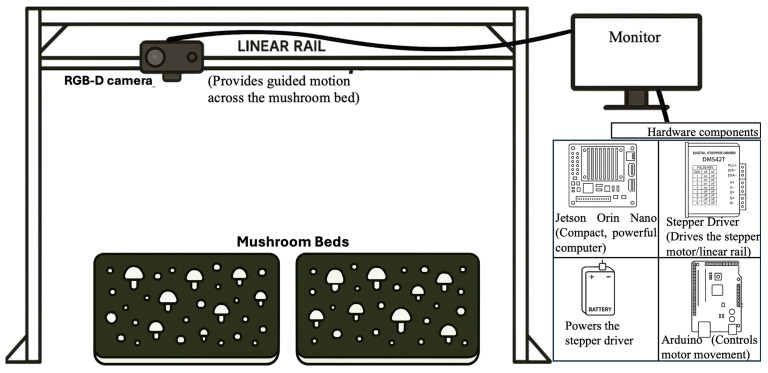
Overview of the imaging setup used for data collection. A stereo RGB-D camera is mounted on a linear rail above the mushroom beds, with supporting hardware (Arduino motor controller, stepper driver, Jetson Orin Nano, and power supply) used for motion control and image acquisition.

Data were collected using a rail-mounted imaging system designed to capture high-resolution RGB and depth information across full white button mushroom beds. The imaging setup is shown in Figure 1. A stereo camera (Zed Mini, StereoLabs, San Francisco, CA, USA) was rigidly mounted on a 1000 mm linear rail and positioned directly above two mushroom beds (571.5 mm × 444.5 mm × 241.3 mm). The camera specifications include a native resolution of 2688 × 1520 pixels, a field of view of 102° (H) × 57° (V) × 118° (D), and an f/2.0 aperture. The camera was oriented downward at a fixed height of 749 mm to ensure consistent geometry and illumination across acquisitions. Motion along the rail was controlled using a stepper motor system driven by an Arduino controller (Shenzhen Elegoo Technology Co., Ltd., Shenzhen, China), while image acquisition and storage were handled by an NVIDIA Jetson Orin Nano (NVIDIA Corporation, Santa Clara, CA, USA).

Depth sensors achieve the most accurate measurements when the target occupies a large portion of the field of view. Because the mushroom beds exceeded the camera’s field of view, capturing the entire bed from a single position would have reduced spatial resolution and degraded depth quality, particularly along cap boundaries. To preserve fine geometric detail, images were therefore captured at multiple overlapping positions along the rail. Specifically, 17 RGB–D frames were acquired at each time point, spaced 60 mm apart, providing substantial overlap between consecutive frames.

Images were collected every 10 min across one growth cycle within a period of four days (18–21 March 2025), totaling ~21.2 h of acquisition time (~5 h per day on average). At each time point, the 17 partially overlapping frames were combined into a single mosaic representing the full mushroom bed. This stitching process was repeated for every acquisition, producing a total of 128 stitched mosaics, which served as the inputs for all subsequent segmentation and tracking analysis.

For acquisition and processing, the camera was configured to output frames at 1280 × 720 pixels. After stitching, the resulting mosaics measured approximately 1280 × 1634 pixels and were cropped to a final size of 615 × 988 pixels to isolate the region of interest corresponding to the mushroom beds. Unless otherwise stated, all references to RGB images or depth images in this study refer to these stitched mosaics rather than individual raw frames. Spatial resolution was estimated using a calibration checkerboard with 50.8 mm squares, yielding approximately 0.925 pixels per millimeter at an imaging height of 749 mm.

Because consecutive frames captured with substantial overlap, an image stitching step was required to combine them into a continuous mosaic. A custom template matching algorithm was applied to both RGB and depth images. For each pair of consecutive frames $I_{i}$ and $I_{i+1}$, a horizontal strip of 100 pixels from the bottom of *I_i_* was extracted as a template *T*. This region is expected to reappear near the top of $I_{i+1}$. Both *T* and *I_i_*_+1_ were converted to grayscale in OpenCV using cv2.cvtColor function. Template matching was then performed in OpenCV with cv2.matchTemplate function, employing the normalized cross-correlation method (TM_CCOEFF_NORMED) [22]. The similarity score $R\left(y\right)$ at the vertical location y in $I_{i+1}$ was computed as

(1) $$\begin{array}{c} R\left(y\right)=TM\left(T,I_{i+1},y\right) \end{array}$$

where $TM\left(\cdot \right)$ denotes the OpenCV template-matching operator [23]. The optimal alignment, $y_{offset}$ was determined as

(2) $$\begin{array}{c} y_{offset}=\arg\underset{y}{\max}\;R\left(y\right) \end{array}$$

**Figure 2:**
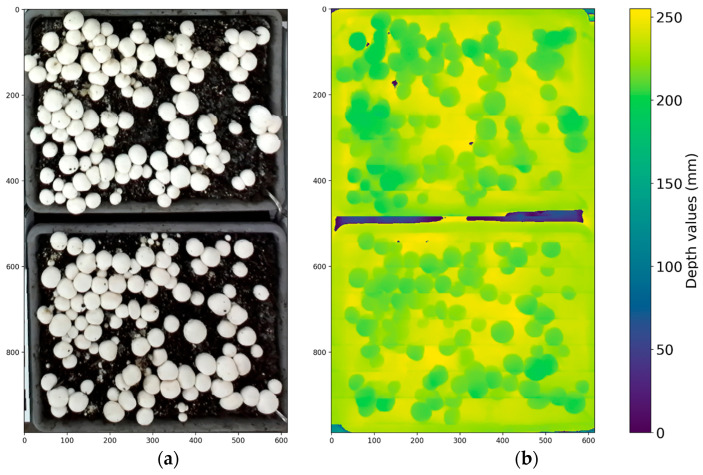
Stitched RGB image (**a**) and corresponding stitched depth map (**b**) of the two mushroom beds. The depth image is visualized using a pseudo-color scale where yellow indicates regions closer to the camera and blue/purple represents farther surfaces or invalid depth values.

The effective vertical overlap between frames was then computed as

(3) $$\begin{array}{c} h_{overlap}=h_{t}+y_{offset}\; \end{array}$$

(4) $$\begin{array}{c} overlap\;ratio=\frac{h_{overlap}}{H}\; \end{array}$$

where $h_{t}$ = 100 pixels is the template height, $y_{offset}$ = 561 pixels, and *H* = 720 pixels is the image height. Under the experimental setup, the resulting overlap ratio was approximately 91.8%. The non-overlapping portion of image *I_i_*_+1_ was appended to the mosaic to produce a composite image with uniform resolution across all images. The corresponding depth mosaic was constructed by applying the same RGB-derived vertical offset to the depth frames, ensuring that the RGB and depth mosaics share identical stitched coordinates and remain spatially aligned. Figure 2 shows an example of a stitched RGB mosaic and its corresponding depth map. In the present study, depth images were not manually annotated separately. The RGB and depth images were captured using a registered RGB–D sensor, producing spatially aligned images with identical resolution and pixel-to-pixel correspondence in the same coordinate frame.

### 2.2. Depth-Guided Image-Processing Pipeline for Mushroom Detection and Segmentation (RD-GuideNet)

**Figure 3:**
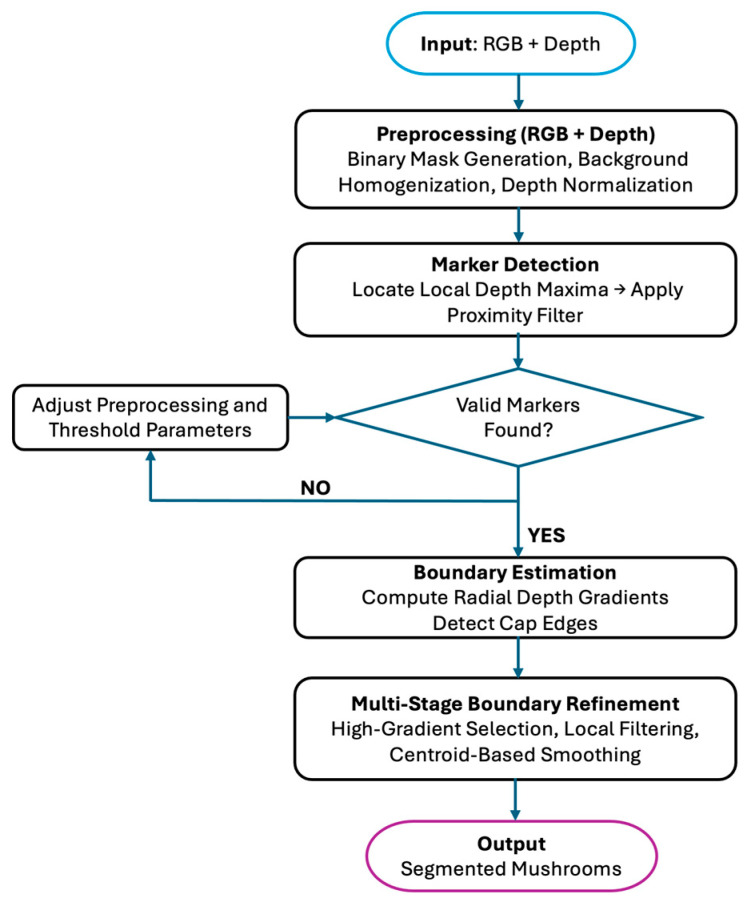
A Flowchart of RD-GuideNet for mushroom detection and segmentation.

The RD-GuideNet pipeline combines color and geometric information from RGB and depth images to improve mushroom detection and boundary segmentation, particularly in dense or overlapping clusters. The overall workflow (Figure 3) consists of four primary stages: preprocessing, marker detection, boundary estimation, and multi-stage refinement.

#### 2.2.1. Image Preprocessing and Background Homogenization

**Figure 4:**
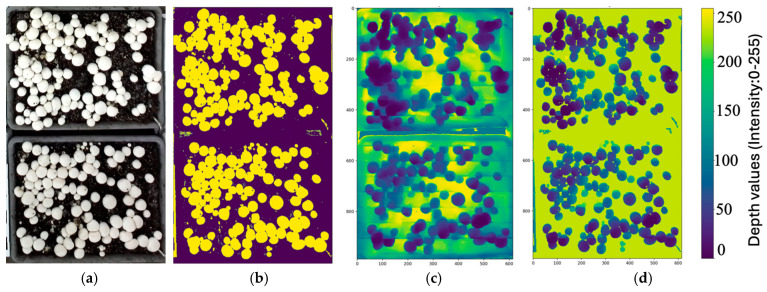
Preprocessing workflow for mushroom image segmentation. (**a**) Original RGB image; (**b**) Binary mask after Otsu thresholding; (**c**) Depth image before background homogenization; (**d**) Depth image after homogenization and normalization. The color bar represents normalized depth intensity values ranging from 0 to 255 (unitless, scaled for visualization and downstream processing).

To create uniform image quality and minimize depth noise, all RGB and depth images were preprocessed before segmentation. Each RGB image (Figure 4a) was converted into a binary mask using Otsu’s thresholding method to separate foreground mushrooms from the compost background (Figure 4b). This binary mask was mapped onto the corresponding depth image (Figure 4c) to identify background pixels. For all background pixels, the mean background depth was computed and reassigned to create a uniform planar surface, reducing artifacts caused by irregular compost texture. After background homogenization, the depth image was normalized using min-max scaling to a range of 0–1 (Figure 4d), resulting in a smoother, more uniform background with clearly defined geometric features in the mushroom.

#### 2.2.2. Marker Detection from Depth Peaks

**Figure 5:**
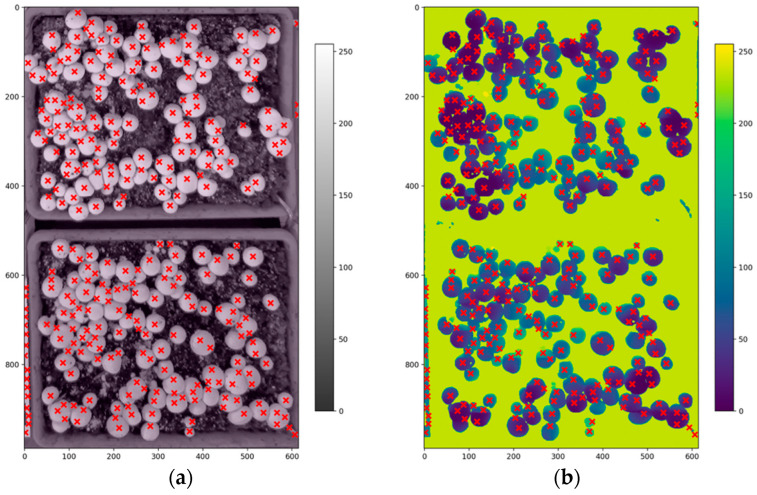
Example of detected markers overlaid on the (**a**) grayscale RGB mosaic and (**b**) depth mosaic. Red crosses represent the final consolidated markers, corresponding to local depth maxima that approximate the apex of each mushroom cap. These markers serve as anchor points for subsequent radial boundary extraction in the RD-GuideNet segmentation pipeline.

Potential mushroom centers were identified directly from the depth image by locating local elevation maxima. A Gaussian-weighted 5 × 5 sliding window with a stride of 3 was applied across the depth image to emphasize central high-elevation pixels while suppressing shallow or noisy variations on the compost surface. The resulting response surface was smoothed using bilateral filtering to eliminate small, spurious peaks caused by surface irregularities. While this smoothing may slightly remove small or isolated peaks, many small mushroom instances still remained detected, and the dominant mushroom structures were largely preserved. Connected Component Analysis (CCA) was performed to isolate contiguous high-elevation regions. From each region, the pixel with the greatest elevation was selected as a potential marker representing the mushroom center. Because mushroom caps can exhibit texture variations or slightly irregular shapes, a single cap may produce multiple markers. To avoid redundant detections in rough or uneven caps, a proximity filter removed markers that were closer than a defined Euclidean distance threshold (18 pixels). Additional markers that did not correspond to distinct mushroom centers could still arise at this stage. However, these markers were merged or removed in subsequent stages. The final marker sets are illustrated in Figure 5.

#### 2.2.3. Boundary Estimation Through Radial Gradient Analysis

**Figure 6:**
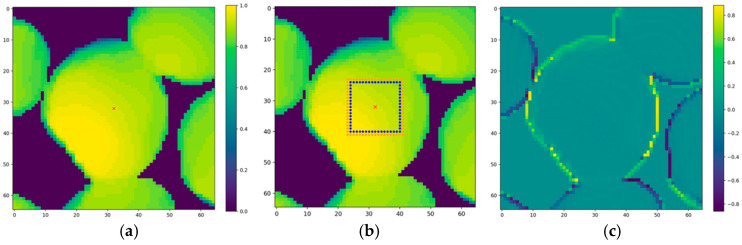
Illustration of the gradient computation used for boundary estimation. (**a**) Depth patch showing the detected center marker (red cross) for the mushroom, (**b**) marker location and the inner (blue) and outer (red) square boundary layers used to compute outward depth differences, and (**c**) resulting gradient map, where high-gradient regions indicate the transition between the mushroom cap and the surrounding background.

After marker detection, the algorithm estimated each mushroom’s boundary by analyzing depth gradients progressing outward from each detected marker (Figure 6a). Because mushroom caps exhibit a gradual decrease in height from the center toward the compost surface, these depth gradients provide reliable geometric cues for edge localization. For each marker, a 23 × 23 patch was extracted from the depth image and normalized using min–max scaling to preserve relative elevation differences. The patch size was empirically chosen to accommodate the largest mushrooms in the dataset. Within each patch, square boundaries were incrementally expanded around the marker (Figure 6b). Each outline expanded outward by one pixel per iteration, indexed as *r* = 1, 2, …, 11.

At each expansion step, the algorithm compared depth values between two consecutive square boundaries. The depth gradient for each pixel p∈B(r) was computed relative to its 8-connected neighbors on the next outer boundary B(r+1). The mean of these pixelwise gradients across all K boundary pixels defined the radial gradient magnitude G(r) (Equation (5)). The operator max{0, ⋅}  suppresses negative gradients caused by adjacent overlapping mushrooms, provided that only descending height transitions contribute to the final gradient magnitude.

(5) $$G\left(r\right)=\frac{1}{K}\sum_{k=1}^{K}max\left\{0\;,\;\;\;\frac{1}{M}\sum_{q\in N_{out}(p_{k})}D\left(p_{k}\right)-D(q)\right\}$$

where *r* represents the index of the current inner boundary layer; *K* is the number of pixels $p_{k}$ on that inner boundary B(r); *M* is the number of outer neighbors (typically 8); $N_{\mathrm{out}}(p_{k})$ denotes the 8-connected outer neighbor set of pixel $p_{k}$; and D(⋅) represents the depth image value.

The resulting gradient map (Figure 6c) highlights high-gradient regions corresponding to the transition between mushroom caps and the surrounding background, providing a precise geometric boundary for segmentation.

#### 2.2.4. Separation of Overlapping Mushrooms

**Figure 7:**
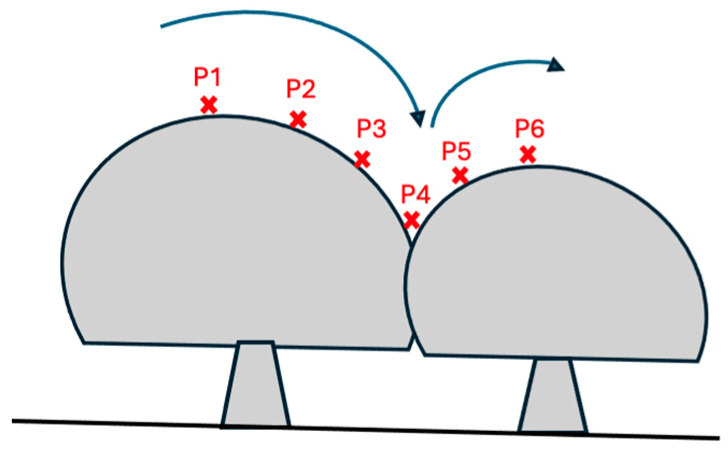
Illustration of how the algorithm distinguishes between cojoined mushrooms. As the radial scan moves outward from point P1 toward P3, depth values decrease until reaching the true edge of the first mushroom (P4). Beyond this point, the depth begins to increase again (P5–P6) due to the adjacent mushroom. This increase indicates the boundary, enabling the algorithm to accurately separate the two mushrooms.

**Figure 8:**
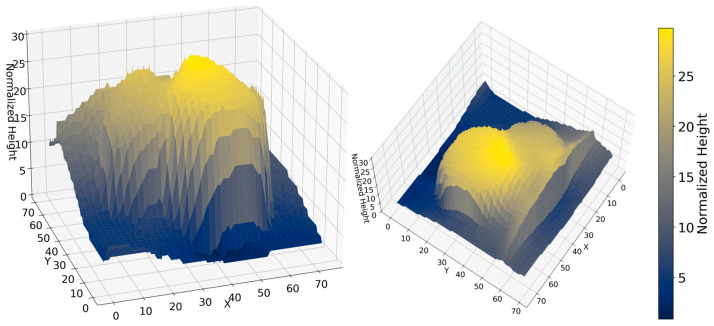
3D surface visualization of the normalized depth/height map for two cojoined mushrooms shown from two viewing angles. The two domes correspond to the mushroom caps, while the valley region between peaks highlights the local minimum associated with the boundary between adjacent mushrooms. The color bar indicates normalized height.

White button mushrooms grow in dense clusters where caps overlap or touch, producing smooth depth transitions that can cause multiple mushrooms to merge into a single segment. For an isolated mushroom, the depth profile decreases monotonically as the algorithm progresses radially outward from the center marker and eventually stabilizes at the compost surface. In contrast, when two mushrooms overlap, the outward depth profile decreases until it reaches the edge of the first cap, then increases again as it encounters the adjacent cap (Figure 7). To detect such an overlap, the algorithm examines the radial depth gradient profile G(r) (Equation (5)) for each marker and locates inflection points where the gradient changes from negative to positive. This transition indicates that the depth has started to rise again, signifying contact with a neighboring cap. The corresponding radius is marked as the boundary limit of the current mushroom. The boundary detection described in Figure 7 is further visualized in Figure 8, where the 3D depth surface shows a valley between the two caps.

#### 2.2.5. Multi-Stage Boundary Refinement and Smoothing

The raw boundary points obtained from radial scanning often contain noise, spikes, and distortions caused by surface irregularities, minor marker misplacement, or interference from neighboring mushrooms. The goal of the refinement is not to redefine the boundary, but to clean and stabilize it by removing spurious points, suppressing leakage into adjacent mushrooms, and recovering a smooth contour. RD-GuideNet performs this refinement through three sequential filtering passes.

**Figure 9:**
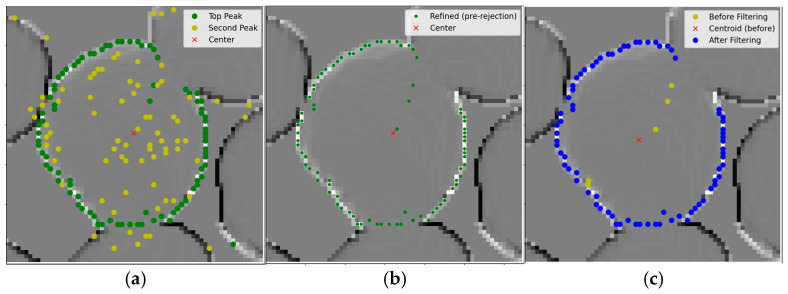
Multi-stage boundary refinement process for RD-GuideNet. (**a**) First-pass candidate identification—green points mark primary high-gradient responses, yellow points indicate secondary candidates retained for later refinement; (**b**) Second-pass local consistency filtering—green points show the boundary after removal of locally inconsistent or spiky points; (**c**) Third-pass centroid-based global adjustment—yellow points represent outliers removed beyond 2.5 standard deviations, while blue points show the final smoothed contour.

- First Pass—High-Gradient Boundary Candidate Selection: Using the gradient magnitudes computed in Section 2.2.3, the first refinement pass identifies the most likely boundary points. For each marker, the algorithm selects the top two gradient peaks (Figure 9a), corresponding to sharp transitions between the mushroom cap and its surroundings. The highest peak is treated as the primary boundary candidate, while the second highest is retained as a backup for potential use in later refinement. These peaks represent the most prominent geometric edges in the depth profile and serve as initial boundary estimates.
- Second Pass—Local Consistency Filtering: Although high-gradient peaks reliably indicate boundary locations, isolated spikes can still appear due to noise or abrupt surface changes. To remove these outliers, each boundary point is compared with its ten immediate neighbors (five on each side along the boundary curve). The algorithm computes the mean radial distance of these neighbors from the marker. If the point’s distance deviates by more than 10 pixels from this local mean, it is rejected. This step enforces local smoothness and eliminates sharp outliers (Figure 9b).
- Third Pass—Centroid-Based Global Adjustment: After local smoothing, some boundary points may still be unevenly distributed around the mushroom center, causing the contour to appear slightly offset or irregular in shape. To correct this, a global adjustment is applied. The centroid of all remaining boundary points is computed, followed by the mean radial distance from this centroid. Any boundary point whose distance deviates by more than 2.5 standard deviations from the mean is removed. This step eliminates residual outliers (yellow points in Figure 9c) and produces a well-centered, uniformly shaped final contour (blue points).
- The final output consists of cleaned point coordinates tracing the boundaries of the detected mushrooms. These coordinates were used to construct a convex hull, which was then used to generate the mushroom segmentation mask. The RGB image was converted to grayscale and binarized into a black-and-white image using Otsu thresholding, with background regions (black) excluded and only foreground regions (white) retained.

### 2.3. Multi-Frame Tracking Based on RD-GuideNet

While RD-GuideNet provides accurate per-frame instance masks, analyzing mushroom growth requires associating individual mushrooms across sequential frames. The tracking module assigns a consistent identifier (ID) to each mushroom so that its size, shape, and development can be monitored throughout the growth cycle. Although the tracking operates independently of RD-GuideNet, it uses the instance masks and depth images generated by RD-GuideNet as inputs to track each mushroom through time.

#### 2.3.1. Overlap Resolution via Depth-Guided Watershed

**Figure 10:**
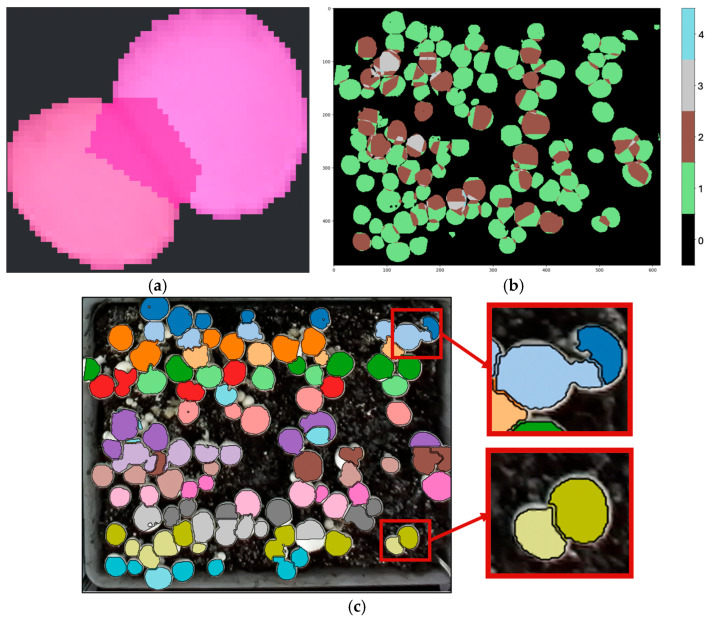
Illustration of pixel coverage count used for overlap detection. (**a**) Overlapping instance masks produced by RD-GuideNet, (**b**) Coverage count map showing pixels claimed by multiple instances (highlighted in brown), and (**c**) Result after depth-guided watershed separation, showing successful splitting of overlapping mushrooms into distinct instances (bottom inset) and an example failure case with an incorrect split (top inset).

**Figure 11:**
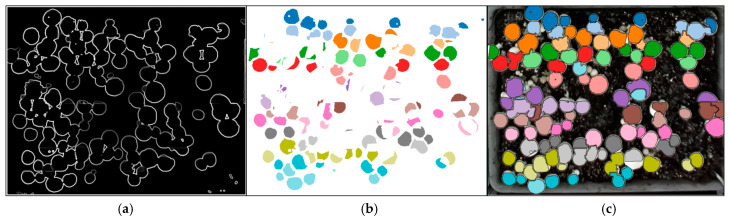
Illustration of the depth-guided, marker-controlled watershed used for separating overlapping mushrooms. (**a**) Ridge map generated by Sobel operator emphasizing cap boundaries, (**b**) Interior seeds assigned to each mushroom instance within confidently labeled regions, and (**c**) Final watershed result showing distinct instance regions separated along ridge lines.

From each instance mask, a coverage count is computed that represents the number of instances claiming each pixel (Figure 10a). Coverage count is computed by scanning each pixel in the image and counting how many mushroom instance masks include that pixel as part of a mushroom instance. Pixels with a coverage count of 1 are non-overlapping and retain their existing labels. Pixels with a coverage count of more than one indicate overlapping regions that must be resolved, since each pixel should belong to only one mushroom (Figure 10b). To separate overlapping instances, a depth-guided, marker-controlled watershed algorithm was applied to suppress noise using depth image. A Sobel operator was then used to compute the gradient magnitude to create a ridge map that emphasized cap boundaries. For each mushroom, an interior seed was defined from confidently labeled interior pixels. The watershed then grows these seeds only within the overlapping area, while the ridge map halts growth at true boundaries (Figure 11). This process ensures that every pixel in the overlap is assigned to exactly one mushroom. After minor morphological cleanup (hole filling and speck removal), the algorithm produces a resolved label image $L_{t}$ for frame *t*, where the background is labeled 0 and each mushroom has a unique instance ID.

#### 2.3.2. Temporal Association Across Frames

**Figure 12:**
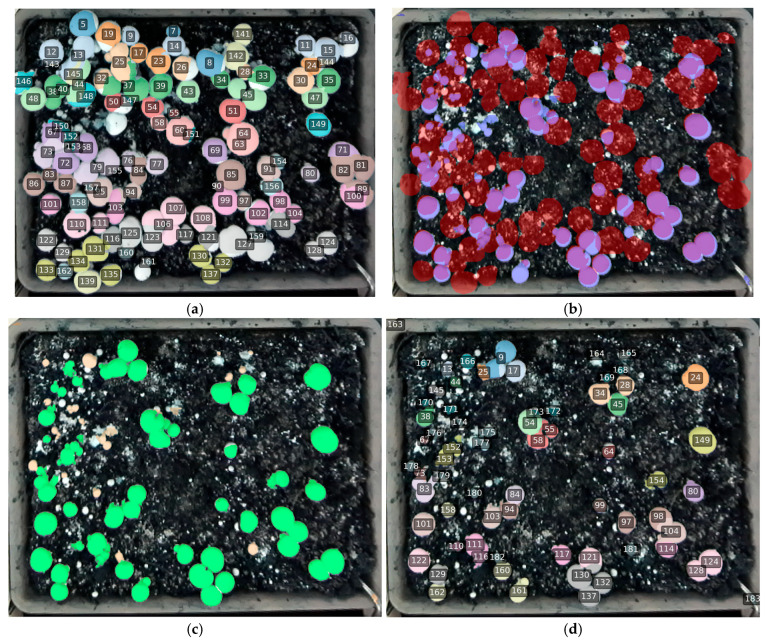
Illustration of temporal association using IoU-based Hungarian matching. (**a**) Mushrooms in frame t − 1 with global IDs, (**b**) Mushrooms in frame t showing overlap between consecutive frames, (**c**) IoU matrix example: cells with IoU ≥ τ (green) are accepted matches, and (**d**) Final ID updates: matched instances inherit previous IDs; new instances receive new IDs; unmatched old instances are terminated.

To maintain consistent IDs through time, resolved instances were linked between consecutive frames $L_{t-1}$ and $L_{t}$. Pairwise Intersection-over-Union (IoU) scores were computed between all instance masks across the two frames. A one-to-one correspondence was then established using the Hungarian matching algorithm [24], which optimized global matching based on IoU. Matches were accepted only when IoU ≥ τ (set to 0.1), a predefined threshold based on experimentation that prevented erroneous links caused by appearance, disappearance, or partial occlusion. Matched instances inherited their previous global ID. Unmatched new instances received new IDs. Unmatched previous instances were marked as inactive and removed if they remain unmatched for subsequent frames (Figure 12). Following this procedure, a tracked label map $\hat{L_{t}}$ is generated by replacing local instance IDs in $L_{t}$ with their assigned global IDs.

### 2.4. Benchmarking with Yolo-Based Deep Learning Models

The YOLOv8-seg [25] and YOLOv11-seg [26] models were selected for comparison because they are widely used for agricultural detection/segmentation and are high-performing instance-segmentation baselines with readily available implementations, enabling a practical benchmark against RD-GuideNet. Both models were trained on the preprocessed RGB patches described in Section 2.1. A total of 12 RGB images were utilized for the segmentation task, with a resolution of 615 × 988 pixels. Each original image (615 × 988 pixels) was divided into 256 × 128 patches to maintain small-cap visibility and local spatial context for dense regions. Training with different patch sizes showed that 256 × 128 provided the best balance between context retention and edge resolution at the network’s working scale. Across the 12 images, a total of 4361 mushroom instances were manually annotated using Roboflow annotation platform [27] where each mushroom was labeled using polygon tool. The final dataset included 4361 annotated mushrooms with 3445 used for training and 916 for testing. Both YOLOv8-seg and YOLOv11-seg models were initialized from pretrained weights using an input image size of 256 × 256 pixels, the Adam optimizer (learning rate = 0.01), batch size = 32, and 50 training epochs. After the initial training phase, YOLOv8-seg was further trained from the best checkpoint for an additional 30 epochs, whereas YOLOv11-seg was trained for 20 additional epochs. For multi-frame instance tracking, the YOLOv8-seg/YOLOv11-seg models were integrated with the ByteTrack multi-object tracker [28]. Although mushrooms remain stationary, their size and appearance change over time, and they often overlap in dense clusters. Additionally, mushrooms may be removed during harvesting, resulting in object disappearance. ByteTrack enables consistent identity assignment across time-lapse images and ensures proper handling of object appearance and disappearance, supporting reliable monitoring of individual mushroom development and harvesting events. For each frame, the YOLO model produced mushroom instance masks, and low-confidence detections were discarded. During inference and tracking, the trained models were applied directly to full stitched frames using an adjusted inference resolution enabling segmentation and tracking across the entire image while maintaining consistency with trained model scale. ByteTrack maintained consistent identities across consecutive frames by matching detections to existing tracks using a two-stage association: (1) a high-confidence pass to establish reliable matches, followed by (2) a lower-confidence pass to recover missed associations and reduce track fragmentation. Matched detections inherited their previous IDs, while new detections above the new-track threshold initiated new IDs. Unmatched tracks were retained for several frames to bridge short occlusions before termination. Table 1 shows the parameters used by the YOLO tracking algorithm.

### 2.5. Evaluation Metrics and Comparative Analysis

To evaluate overall system performances, three components were assessed: detection, segmentation, and multi-frame tracking.

#### 2.5.1. Detection Metrics

Detection performance was evaluated using an intersection-over-union (IoU) threshold of 0.7 to determine true positives (TP). The IoU between the predicted region $M_{pred}$ and ground-truth region $M_{gt}$ was computed as shown in Equation (6).

(6) $$\begin{array}{c} IoU=\frac{\left|M_{pred}\cap M_{gt}\right|}{\left|M_{pred}\cup M_{gt}\right|} \end{array}$$

Precision, recall, and F1-score were calculated using the standard formulas such as:

(7) $$\begin{array}{c} Precision=\frac{TP}{TP+FP} \end{array}$$

(8) $$\begin{array}{c} Recall=\frac{TP}{TP+FN} \end{array}$$

(9) $$\begin{array}{c} F1=\frac{2\times Precision\times Recall}{Precision+Recall} \end{array}$$

False positives (FPs) were defined as predicted instances that did not match any ground-truth instance, whereas false negatives (FNs) were ground-truth instances that were not matched by any predicted instance.

#### 2.5.2. Segmentation Metrics

Segmentation performance was evaluated at the pixel level by comparing the predicted mushroom masks with the corresponding ground-truth annotations. Unlike object-level detection metrics, pixel-level evaluation assesses the spatial accuracy of the segmented regions and is particularly important in dense production beds where mushrooms frequently overlap or exhibit irregular boundaries. In instance segmentation, each individual mushroom is assigned a separate mask, and evaluation requires comparing predicted masks with their corresponding ground-truth masks based on spatial agreement. Pixel-wise true positives (TPs) were defined as pixels labeled as mushroom in both the predicted and ground-truth masks. Pixel-wise false positives (FPs) were pixels incorrectly labeled as mushroom in the prediction but not in the ground-truth, while pixel-wise false negatives (FNs) were mushroom pixels present in the ground-truth but missed by the model. Using these definitions, the same standard formulas for precision, recall, and F1-score (Equations (7)–(9)) were applied to quantify segmentation accuracy.

#### 2.5.3. Tracking Metrics

Tracking performance was evaluated to measure whether mushroom instances maintain consistent identifiers across consecutive frames. After segmentation, each mushroom was assigned a unique identifier (ID), which was monitored throughout the image sequence to assess the accuracy and stability of temporal associations. In this study, a track was defined as the sequence of detections of a single mushroom across consecutive frames in which it appeared. For evaluation, 128 stitched mosaic frames containing 298 individual mushroom tracks were manually inspected. Each track was reviewed to determine whether the same mushroom retained a consistent ID across all frames without identity swaps or interruptions. Tracking consistency was computed as the proportion of correctly maintained tracks as defined in Equation (10).

(10) $$\begin{array}{c} Tracking\;consistency\;\left(\%\right)=\frac{N_{consistent}}{N_{inspected}}\times 100\; \end{array}$$

where $N_{consistent}$ denotes the number of tracks with no ID errors, and $N_{inspected}$ represents the total number of evaluated tracks (298 in this study).

## 3. Results and Discussions

### 3.1. Detection and Segmentation Performance

Table 2 presents the quantitative comparison of detection and segmentation performance across RD-GuideNet, YOLOv8-seg, and YOLOv11-seg. In RD-GuideNet, a mushroom was considered successfully detected if at least one marker fell within its manually annotated cap region. When multiple markers appeared on the same mushroom, they were merged before instance-level evaluation since they captured the same region and were handled in the overlap resolution stage. However, these additional markers were retained during segmentation to refine local boundary definition. For segmentation evaluation, false positives and false negatives were computed at the pixel level, based on the overlap between predicted and ground-truth masks, independent of the number of markers.

RD-GuideNet achieved high precision for both detection (0.95) and segmentation (0.98), confirming that its depth-guided geometry enables highly reliable identification of true mushroom instances while minimizing false positives. This precision advantage arises from the model’s reliance on 3D structural cues rather than 2D texture similarity. By explicitly encoding the cap curvature and height difference from the compost surface, RD-GuideNet effectively suppresses misclassification of compost clumps or irregular lighting artifacts that frequently confuse RGB-based detectors.

RD-GuideNet’s recall (0.93) was slightly higher than that of the YOLO models (0.91 and 0.92), reflecting a more balanced precision-recall trade-off. The remaining missed cases were primarily associated with small, partially occluded, or low-elevation mushrooms that generated weaker curvature gradients, which were easily suppressed during depth smoothing and thresholding. Consequently, such mushrooms remain undetected even though they are visually identifiable in the RGB image.

**Figure 13:**
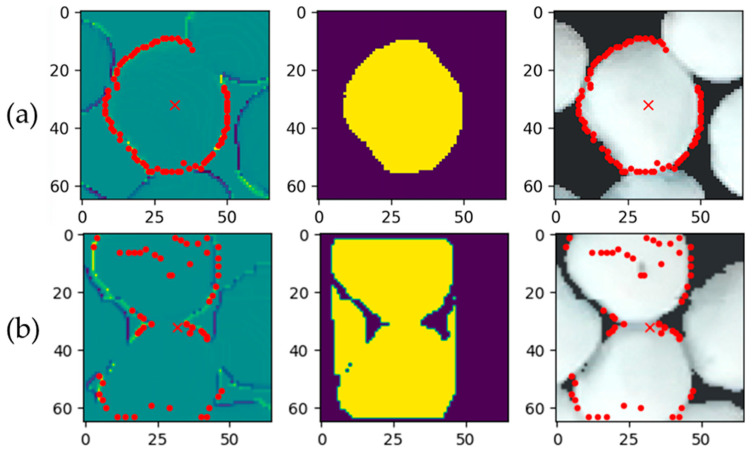
Example images of segmentation results. The left column shows boundary detection in the gradient image, the middle column shows the resulting segmentation mask, and the right column shows boundary detection overlaid on the RGB image for (**a**) an accurately segmented mushroom and (**b**) an incorrectly segmented case (merged multiple mushrooms into a single mask). The red “x” depicts the marker of the mushroom and the red dots represent the boundary of the mushrooms.

Representative examples of RD-GuideNet’s segmentation outcomes are shown in Figure 13. In the correctly segmented case (Figure 13a), the boundary points accurately trace the mushroom cap perimeter in both the gradient and RGB images, resulting in a clean and well-defined mask. In contrast, Figure 13b shows a typical mis-segmentation scenario that contributes to RD-GuideNet’s lower segmentation recall. When adjacent caps are in contact, and the depth transition between them is shallow, the boundary points near the junction become sparse or ambiguous. Consequently, the watershed process merges neighboring caps into a single component, causing one instance to be missed in detection. These merged-mask cases were the primary source of recall loss, as the mushrooms were visually present but not recognized as separate objects. Such errors predominantly occurred in densely clustered or partially occluded regions, rather than from background confusion or leakage.

**Figure 14:**
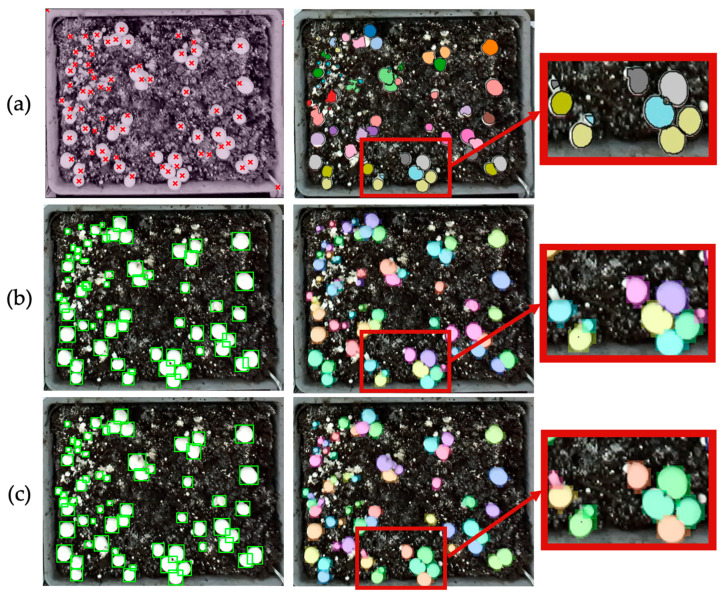
Comparative results of mushroom detection and segmentation using (**a**) RD-GuideNet, (**b**) YOLOv8-seg, and (**c**) YOLOv11-seg. Red crosses indicate detected mushroom centers using RD-GuideNet, green boxes represent detection bounding boxes from the YOLO-based models, and colored masks represent segmented instances. Among 74 total mushrooms, RD-GuideNet detected 59 and YOLO-based models detected 63. Enlarged boxes show that RD-GuideNet produces smoother and more circular boundaries that closely match the true mushroom cap shapes, while YOLO-based models exhibit irregular and merged contours, particularly in dense clusters.

YOLOv8-seg and YOLOv11-seg achieved lower detection recall (0.92 and 0.91, respectively) and overall detection F1-scores of 0.95 and 0.94 on the same dataset. Their segmentation performance was lower (F1 ≈ 0.86–0.88), reflecting a tendency to generate imprecise boundaries, particularly in dense clusters or partially occluded regions. These differences arise primarily from how the models interpret visual evidence. YOLO networks rely solely on RGB appearance cues and learn discriminative shape priors from annotated examples, enabling them to recognize mushrooms even when depth-based geometric cues are weak or ambiguous. As a result, they successfully localize small or shaded mushrooms that RD-GuideNet may miss. Yet, because their learned features are appearance-driven rather than geometry-aware, the resulting masks often exhibit irregular edges, shape distortion, or partial merging between adjacent caps when texture contrast is low. As illustrated in Figure 14, YOLOv8-seg and YOLOv11-seg correctly detect more instances overall but produce polygonal and merged masks in tightly packed areas. The bounding boxes (green) often overlap multiple caps, and the corresponding color masks blend neighboring mushrooms into irregular shapes. RD-GuideNet, by contrast, delineates each mushroom with smoother, more circular contours that better approximate its physical morphology. This morphological accuracy is crucial for downstream phenotyping, where precise boundary definition directly affects volume estimation, growth-rate analysis, and robotic grasp planning. The superior geometric fidelity of RD-GuideNet provides that cap area and centroid locations correspond more faithfully to real mushroom structures, even at the cost of missing a few occluded instances.

These results indicate a fundamental trade-off between completeness and morphological accuracy. YOLO-based models favor inclusivity of detecting nearly every visible object, whereas RD-GuideNet prioritizes structural realism and segmentation precision. In practice, YOLOv8-seg and YOLOv11-seg may be preferable for large-scale yield estimation or scouting applications where broad coverage is critical, while RD-GuideNet provides the reliability and geometric consistency needed for robotic harvesting and growth modeling.

### 3.2. Comparative Tracking Performance of RD-GuideNet and YOLO Models

**Figure 15:**
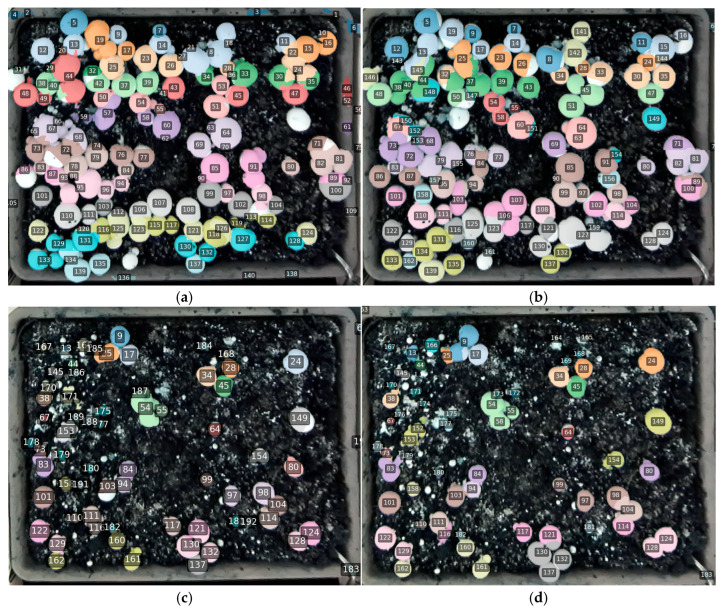
Example of RD-GuideNet-based multi-frame tracking of white-button mushrooms across sequential frames within the same bed. (**a**) Initial frame showing assigned track IDs; (**b**) frame captured 10 min later, where most mushrooms retain identical IDs despite minor changes in cap size, pose, and local crowding, demonstrating robust temporal association; (**c**,**d**) frames acquired post-harvest, where IDs corresponding to removed mushrooms are absent, while remaining mushrooms preserve their prior identities. The consistent color-coded IDs and well-preserved boundaries confirm RD-GuideNet’s ability to maintain long-term identity continuity, even under occlusion and partial harvesting.

**Figure 16:**
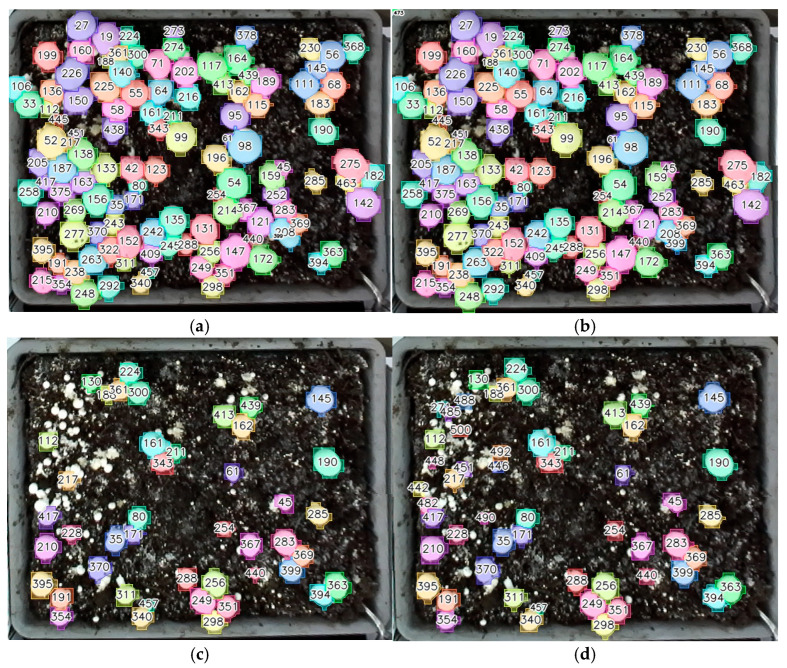
Multi-frame tracking of white-button mushrooms using YOLOv8-seg across sequential frames from the same bed. (**a**) Initial frame with assigned track IDs; (**b**) frame acquired 10 min later showing overall ID stability and broad coverage across dense regions, though some touching caps appear merged; (**c**,**d**) frames captured post-harvest, where IDs for removed mushrooms are absent while most remaining instances preserve their previous identifiers. YOLOv8 maintains good temporal linkage for clearly separated caps but occasionally loses or merges instances in crowded areas, leading to inconsistent tracking coverage in later frames.

Figure 15 and Figure 16 compare the temporal tracking behavior of RD-GuideNet and YOLO-based pipelines over consecutive time-lapse frames. Both frameworks successfully maintain unique identities for most mushrooms, demonstrating that geometric and appearance cues can each support temporal association under stable imaging conditions. However, their tracking behaviors diverge in how they handle occlusion, crowding, and gradual morphological changes during growth and harvesting.

RD-GuideNet (Figure 15) produced sharp, well-defined instance boundaries that closely followed cap curvature. These geometrically precise contours contributed to accurate frame-to-frame matching, particularly for isolated or moderately spaced mushrooms. Across 10 min intervals, most instances retained identical identifiers (IDs), even as their sizes or poses changed slightly, confirming RD-GuideNet’s strong temporal stability and low inter-frame drift. After partial harvesting (Figure 15c,d), IDs associated with removed mushrooms disappeared while the remaining mushrooms preserved their prior labels, illustrating the model’s ability to sustain long-term identity continuity and handle dynamic scene changes without cascading tracking errors. Despite these advantages, RD-GuideNet occasionally failed to assign IDs to very small, low-contrast, or occluded mushrooms. Such cases occur when the depth elevation peak is insufficient for marker formation, preventing detection at the initial stage. Each missed detection disrupts the track for that mushroom, leading to temporary disappearance or a new ID in later frames. Additionally, minor boundary shifts from depth noise or threshold variation can lower the inter-frame Intersection-over-Union (IoU) below the association threshold, triggering occasional ID resets. These errors are localized and geometry-dependent, arising mainly from weak depth cues rather than systematic algorithmic drift.

In contrast, YOLOv8-seg tracking (Figure 16) achieved broader coverage, detecting most mushrooms across all frames, including those with minimal height contrast. Because YOLO relies on RGB appearance features and learns from annotated texture-shape patterns, it is less affected by weak elevation signals. This led to smoother frame-to-frame detection continuity and slightly higher track retention in dense areas. However, the trade-off was spatial precision. YOLO masks often exhibited irregular or merged boundaries when adjacent caps overlapped, producing occasional under-segmentation. As seen in the later frames (Figure 16c,d), these merged instances sometimes caused identity leakage, where two mushrooms temporarily shared a single ID or one instance disappeared from tracking altogether.

**Figure 17:**
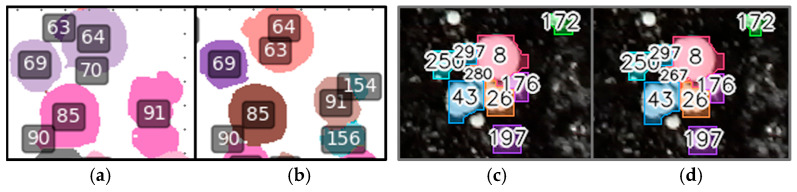
Comparison of ID-switch behavior in RD-GuideNet (**left**) and YOLOv8-seg (**right**). (**a**,**b**) In RD-GuideNet, identity swaps occur when depth gradients are weak or when two caps momentarily merge in the depth map, causing temporary reassignment of track IDs (e.g., 63 ↔ 64). (**c**,**d**) In YOLOv8-seg, ID switches appear in contact regions where adjacent caps share similar texture or illumination (e.g., 280 ↔ 267).

Both RD-GuideNet and YOLOv8-seg occasionally produced ID switches when adjacent mushrooms temporarily merged or changed appearance across frames. Although both frameworks maintained strong overall temporal continuity, the mechanisms underlying these switches reflected their respective sensing domains. Figure 17 provides a focused comparison of ID-switch behavior. In RD-GuideNet (Figure 17a,b), identity swaps occurred when two caps momentarily merged in the depth map or when local elevation gradients weakened, causing brief reassignment of track IDs (e.g., 63 ↔ 64). In YOLOv8-seg (Figure 17c,d), similar ID exchanges appeared in contact regions where neighboring caps shared similar color, texture, or illumination (e.g., 280 ↔ 267).

Based on manual evaluation of 298 mushroom trajectories from 128 stitched mosaics, RD-GuideNet achieved a track-consistency rate of 92.7%, compared with 95.3% for YOLOv8-seg and 94.6% for YOLOv11-seg. Although the numerical gap appears modest, it reflects differences in tracking strategies. RD-GuideNet performs mask-level IoU matching following depth-guided overlap resolution, whereas YOLO models primarily rely on bounding box IoU for temporal association. Because mask IoU is inherently more sensitive to small frame-to-frame boundary variations and local segmentation fluctuations, even minor contour changes can reduce overlap and interrupt identity continuity. In contrast, box IoU is generally more tolerant to such variations, which may contribute to the slightly higher tracking consistency observed with YOLO models. Additionally, RD-GuideNet’s conservative depth-based logic minimizes false positives but increases the likelihood of track breaks when caps are partially occluded or when compost irregularities weaken the geometric signal. YOLO models, conversely, maintain higher continuity by leveraging appearance-based similarity, but at the cost of reduced boundary precision and occasional ID confusion in clusters. These findings reveal a key trade-off between geometric precision and temporal stability. RD-GuideNet’s accurate per-frame segmentation provides highly reliable boundaries valuable for downstream applications such as cap-size estimation, maturity grading, or robotic gripper alignment, where boundary accuracy is critical. YOLO models, while less precise at the pixel level, offer smoother temporal consistency ideal for long-term monitoring or large-scale yield analysis.

In addition to segmentation accuracy and temporal performance, computational resource usage and inference time were evaluated. All experiments were conducted on a laptop equipped with a 2.3 GHz 8–Core Intel Core i9 processor, 16 GB DDR4 RAM (2667 MHz), and integrated Intel UHD Graphics 630 running macOS. Inference time was measured using full stitched mosaic images (~300 mushrooms per image). RD-GuideNet required approximately 29 min per image, corresponding to about 5.8 s per mushroom. Under the same hardware conditions, YOLOv8-seg and YOLOv11-seg combined with ByteTrack required approximately 0.115 s per image. The results highlight a clear difference in computational characteristics between the two approaches. RD-GuideNet performs segmentation through a sequence of depth-guided geometric operations that explicitly refine mushroom boundaries, whereas YOLO-based models perform detection and segmentation through a single forward pass of a convolutional neural network using highly optimized deep-learning frameworks. As a result, YOLO-based models provide significantly higher inference throughput, while RD-GuideNet emphasizes geometric boundary precision. Further improvements in computational efficiency will be pursued through algorithm parallelization and hardware-specific acceleration as part of future system-level development.

### 3.3. Qualitative Insights and Implications for Autonomous Mushroom Harvesting

In addition to quantitative metrics, qualitative inspection of segmentation and tracking outputs revealed complementary behaviors between the geometry-based RD-GuideNet and appearance-driven YOLO models. RD-GuideNet consistently produced sharply delineated and physically accurate mushroom contours, closely matching true cap perimeters even under varying illumination or surface texture. Its reliance on 3D curvature and depth contrast allowed it to suppress false detections from compost artifacts and lighting reflections. However, in regions where elevation gradients weakened, such as small, low-contrast, or partially occluded mushrooms, the algorithm occasionally failed to generate a marker, resulting in missed detections or fragmented tracks.

Conversely, YOLOv8-seg and YOLOv11-seg achieved strong instance coverage and stable detections, reflecting the robustness of RGB-based learning to variations in lighting and surface texture. Yet, their segmentation masks exhibited boundary irregularities, occasional overreach into the background, or fusion between adjacent caps in dense clusters. These qualitative behaviors align with the numerical results, and RD-GuideNet excels in boundary precision and shape fidelity, while YOLO models prioritize detection completeness and temporal stability.

These contrasting strengths can be synergistically integrated to meet the requirements of autonomous mushroom harvesting. The observed differences between RD-GuideNet and YOLO-based segmentation models highlight important trade-offs when considering deployment in autonomous mushroom harvesting systems. In dense mushroom production beds, accurate boundary delineation is critical for estimating cap size, determining grasp points, and avoiding unintended contact with neighboring mushrooms. RD-GuideNet achieved higher segmentation precision (0.95) and F1 score (0.93), indicating that predicted masks closely follow the true mushroom contours. Such boundary accuracy can improve geometric measurements such as cap diameter and centroid location, which are essential for reliable grasp planning and collision-free manipulation.

At the same time, detection recall influences harvesting coverage and operational efficiency. Models with lower recall may fail to detect some mushrooms present in the scene, resulting in missed harvesting opportunities. YOLOv8 and YOLOv11 exhibited comparable recall (≈0.91–0.92) while detecting mushrooms across the bed, including those with subtle geometric features. From a harvesting perspective, maintaining sufficient recall helps ensure that a large proportion of harvestable mushrooms are detected during each scanning cycle, thereby reducing missed targets and improving harvesting throughput.

Temporal consistency is another important factor for robotic operation. Stable segmentation across consecutive frames helps maintain consistent estimates of mushroom position and geometry, supporting reliable tracking and motion planning during continuous harvesting. YOLO-based models demonstrated higher temporal stability due to smoother inter-frame predictions, while RD-GuideNet masks may vary slightly because of depth noise or threshold sensitivity in geometric processing. However, RD-GuideNet’s refined boundaries provide more accurate spatial localization once a target is identified, which is particularly beneficial during grasp planning and manipulation.

Computational efficiency also plays an important role in practical robotic deployment. Real-time harvesting systems require perception algorithms that can operate within the computational constraints of embedded platforms typically used in agricultural robots. As shown in Section 3.2, YOLO-based models provide substantially faster inference, enabling rapid scene analysis and candidate identification. In contrast, RD-GuideNet focuses on depth-guided geometric refinement that improves boundary precision but involves additional processing steps. These differences illustrate a trade-off between computational efficiency and geometric segmentation accuracy.

These findings suggest that the two approaches address complementary aspects of robotic harvesting perception. Fast neural detectors can support rapid scene understanding and large-scale candidate identification, while geometry-driven refinement methods provide the precise boundary information required for accurate grasp planning and manipulation. Integrating these capabilities within a unified perception pipeline may therefore enable both efficient detection and high-precision boundary estimation for automated harvesting in dense production environments.

### 3.4. Relation to Prior Work and Benchmarking Context

Prior work most closely related to this study can be grouped into three directions: (1) RGB-only detection/segmentation for counting and yield estimation; (2) 2D RGB time-lapse tracking that associates instances across frames; (3) depth-/3D-assisted methods that incorporate geometric cues for boundary localization or separation of touching objects. RGB-based pipelines, including both traditional feature-based methods (e.g., morphological/edge-based approaches) and recent learning-based instance segmentation, have been widely used for mushroom detection and segmentation [12,13,14,16,17,18,19]. However, performance often degrades in dense production beds where caps touch or overlap and where intra-cap texture variation can fragment masks or cause merge/split errors. Existing mushroom tracking approaches similarly remain predominantly 2D and RGB-based. For example, prior studies have used simplified geometric models (e.g., circle-based propagation) or IoU-based association of instance masks to track mushrooms over time [20,21]. These strategies can perform well when instances are visually separable, but they are sensitive to segmentation noise and can experience identity instability when masks split or merge under frequent occlusions. In parallel, depth/3D sensing has been explored to improve geometric measurement and localization (e.g., depth-based diameter estimation, point-cloud pose estimation), demonstrating the value of geometric cues for visually ambiguous scenes [6,15]. Nonetheless, many depth-assisted studies focus on single-frame processing and do not evaluate the temporal association of individual mushrooms over time in comparable dense-bed settings.

Direct benchmarking against a single end-to-end “state-of-the-art” pipeline is, therefore, limited because to our knowledge prior studies do not simultaneously report RGB–depth-guided instance segmentation and tracking of individual mushrooms over multiple time points in dense production beds under a comparable evaluation protocol. Accordingly, this study benchmarked segmentation against established RGB baselines and reported standard tracking metrics, while using qualitative analyses (e.g., overlap separation examples) to illustrate how depth-guided separation improves boundary delineation and reduces ambiguity in crowded scenes.

Given this context, RD-GuideNet was designed to address the coupled challenges of overlap resolution and temporal consistency. Unlike RGB-only pipelines that rely primarily on appearance cues, the proposed approach explicitly leverages depth information to identify overlap-prone regions and guide instance separation, which is particularly relevant when contacting caps produce ambiguous boundaries in RGB. The resulting geometry-aware instance masks are then used within a tracking workflow to maintain consistent instance identities across sequential frames, aligning segmentation and tracking objectives rather than treating tracking solely as a post hoc association step. In this study, this design emphasis was reflected in improved segmentation performance relative to the evaluated deep learning baselines, while maintaining tracking consistency that was comparable under the same dataset and protocol.

RD-GuideNet was developed for white button mushrooms (*Agaricus bisporus*), which have near-spherical caps and represent the most widely cultivated commercial mushroom variety. The framework leverages depth-based geometric cues tailored to dome-shaped structures, making it well suited for dense production beds of spherical-cap mushrooms. Extension to other crops with substantially different morphologies would require adaptation of certain geometric assumptions and parameters.

## 4. Conclusions

This study introduces RD-GuideNet, a novel depth-guided segmentation and tracking framework that integrates 3D geometric cues for high-precision mushroom detection in dense production environments. Unlike conventional deep learning models that rely solely on RGB appearance cues, RD-GuideNet integrates 3D geometric reasoning through radial depth gradients, concentric boundary estimation, and multi-stage contour refinement. This depth-aware design allows accurate delineation of mushroom caps even in densely packed and visually ambiguous production environments. Another key contribution of this work lies in the extension of this geometry-based framework to multi-frame tracking. By combining marker-controlled watershed segmentation with depth-informed ID assignment, RD-GuideNet maintains temporal consistency of individual mushrooms across sequential frames, an essential step toward continuous growth monitoring and harvest decision-making. Comparative evaluation against YOLOv8-seg and YOLOv11-seg models shows the originality and complementarity of this approach. RD-GuideNet achieved superior contour fidelity and segmentation precision, reflecting its ability to capture physically meaningful contours critical for robotic manipulation (precision = 0.95; recall = 0.93; F1-score = 0.94 for detection; precision = 0.98; recall = 0.89; F1-score = 0.93 for segmentation; track consistency = 92.7%), while YOLO-based models offered higher detection precision (0.97 and 0.99) and smoother temporal stability (≈95% track consistency) through learned RGB appearance features. These results reveal the distinct value of integrating geometric modeling with data-driven models.

Future research will focus on embedding RD-GuideNet within real-time robotic harvesting platforms, optimizing computational efficiency, and exploring hybrid architectures that combine the spatial interpretability of geometry-based models with the adaptability of deep learning networks. By uniting these two paradigms, this study lays the groundwork for precise, reliable, and explainable perception in autonomous agriculture.

## Figures and Tables

**Table 1:** Parameters used for the YOLO tracking algorithms.

Parameter	Purpose	Values
Model	Fine-tuned YOLOv8-seg/YOLOv11-seg weights	best.pt
imgsz	Inference resize	640
conf	Minimum detection confidence	0.35
tracker	Association backend	bytetrack.yaml
match_thresh	Min threshold to accept a track/detection match	0.5–07
track_high_thresh	High-score cutoff for ByteTrack pass-1	0.6–0.7
track_low_thresh	Low-score floor for ByteTrack recovery pass	0.1–0.3
new_track_thresh	Minimum score to start a new track	0.6
track_buffer	Maximum frames to keep an unmatched track alive	15–30

**Table 2:** Comparison of detection and segmentation performance across RD-GuideNet, YOLOv11, and YOLOv8.

Metric	RD-GuideNet	YOLOv11	YOLOv8
Precision (detection)	0.95	0.97	0.99
Recall (detection)	0.93	0.91	0.92
F1-score (detection)	0.94	0.94	0.95
Precision (segmentation)	0.98	0.88	0.91
Recall(segmentation)	0.89	0.83	0.84
F1-score (segmentation)	0.93	0.86	0.88

## Data Availability

The dataset supporting the findings of this study is publicly available in the Mendeley Data repository at https://doi.org/10.17632/8n6nr43gk3.1, accessed on 17 March 2026.
